# KOUNCIL: Kidney-Oriented Understanding of Correcting Ciliopathies

**DOI:** 10.1186/2046-2530-4-S1-P50

**Published:** 2015-07-13

**Authors:** M Stokman, M Oud, J Van Reeuwijk, M Lilien, N Van De Kar, I Nijman, C Gilissen, HY Kroes, E Bongers, N Geijsen, E Kamsteeg, E Cuppen, R Roepman, R Giles, K Renkema, H Arts, N Knoers

**Affiliations:** 1 Medical Genetics, University Medical Center Utrecht, the Netherlands; 2 Human Genetics, Radboud University Medical Center, Nijmegen, the Netherlands; 3 Pediatric Nephrology, Wilhelmina Children's Hospital, University Medical Center Utrecht, Utrecht, The Netherlands; 4 Pediatric Nephrology, Radboud University Medical Center, Nijmegen, the Netherlands; 5 Hubrecht Institute, University Medical Center Utrecht, the Netherlands; 6 Nephrology and Hypertension, University Medical Center Utrecht, the Netherlands

## Objective

Nephronophthisis is an autosomal recessive renal ciliopathy that constitutes the leading monogenic cause of end-stage renal disease in children. The KOUNCIL consortium is a collaboration between the UMC Utrecht, the Radboud UMC Nijmegen and UC London aimed at elucidating the genetic etiology and pathophysiological mechanisms underlying nephronophthisis and identifying drugs that prevent or delay renal insufficiency. Our goal is to improve genome diagnostics, genetic counselling and therapeutic options for nephronophthisis patients.

## Methods

We employ next-generation sequencing to identify novel disease genes in 100 nephronophthisis patients included within the AGORA biobank project. The functional effect of novel mutations is assessed using *in vitro *and *in vivo *models. Genotypic and phenotypic patient characteristics are registered in a nephronophthisis database, facilitating correlation analyses and identification of early phenotypic markers. Newly identified nephronophthisis-genes are incorporated into diagnostic next-generation sequencing panels of ciliary genes. We use a systems-biology approach to identify and functionally characterize nephronophthisis-associated protein modules. Finally, we use high-throughput repurposing screens in zebrafish embryos to identify FDA-approved drugs that halt renal failure.

## Results

With this approach, we expect to uncover the causal mutation in 60-90% of nephronophthisis patients. KOUNCIL members were involved in the recent identification of three novel genes (*IFT172*, *WDR34 *and *WDR60*) for nephronophthisis-related disorders. Clinical guidelines and new diagnostic tools for nephronophthisis are developed and implemented in diagnostics. We expect to identify drugs that can lead to novel therapies for nephronophthisis.

## Conclusion

The KOUNCIL study is designed to advance understanding of renal ciliopathies and improve clinical care for nephronophthisis patients.

